# Tertiary Lymphoid Structures Across Organs: Context, Composition, and Clinical Levers

**DOI:** 10.1111/imr.70063

**Published:** 2025-09-25

**Authors:** Stephane M. Guillaume, Cristian G. Beccaria, Matteo Iannacone, Michelle A. Linterman

**Affiliations:** ^1^ Immunology Program Babraham Institute Cambridge UK; ^2^ Division of Immunology, Transplantation, and Infectious Diseases IRCCS San Raffaele Scientific Institute Milan Italy; ^3^ Vita‐Salute San Raffaele University Milan Italy; ^4^ Experimental Imaging Center IRCCS San Raffaele Scientific Institute Milan Italy; ^5^ Malaghan Institute of Medical Research Wellington New Zealand

**Keywords:** B cells, germinal centers, tertiary lymphoid structures

## Abstract

Tertiary lymphoid structures (TLSs) arise in non‐lymphoid tissues in response to persistent antigen stimulation and chronic inflammation. Spanning organs from lung and liver to meninges, skin, and beyond, TLSs range from loose aggregates of immune cells to fully mature structures containing functional germinal centers (GC). In this review, we provide a comprehensive overview of TLS formation, architecture, and function across diverse tissues, highlighting both shared features and tissue‐specific adaptations. We then explore the clinical relevance of TLS in infections, autoimmunity, cancer, and allergy, emphasizing their dual roles in mediating protective immunity and driving pathology. Finally, we discuss emerging technologies that are transforming our ability to dissect TLSs at high resolution (including spatial multi‐omics, advanced imaging, and digital pathology), enabling mechanism‐guided strategies to modulate TLSs therapeutically. Framing TLSs through the lens of maturation and tissue context provides a foundation for interpreting their clinical associations and for enhancing or dismantling these niches according to need.

## 1 Introduction

In response to infection or persistent inflammation, leukocytes can organize into structured aggregates within non‐lymphoid tissues. These formations, known as tertiary lymphoid structures (TLSs), resemble secondary lymphoid organs (SLOs) such as lymph nodes (LNs) and the spleen in both organization and function. Although conventional germinal centers (GCs), highly specialized sites of B cell clonal expansion and antibody affinity maturation, were identified over a century ago, the concept that TLSs can form *de novo* in peripheral tissues in response to immune activation has only gained broader recognition in recent decades [1]. This evolving understanding of TLS biology has opened new perspectives on their roles in host defense, chronic inflammation, and local immunity.

**FIGURE 1:**
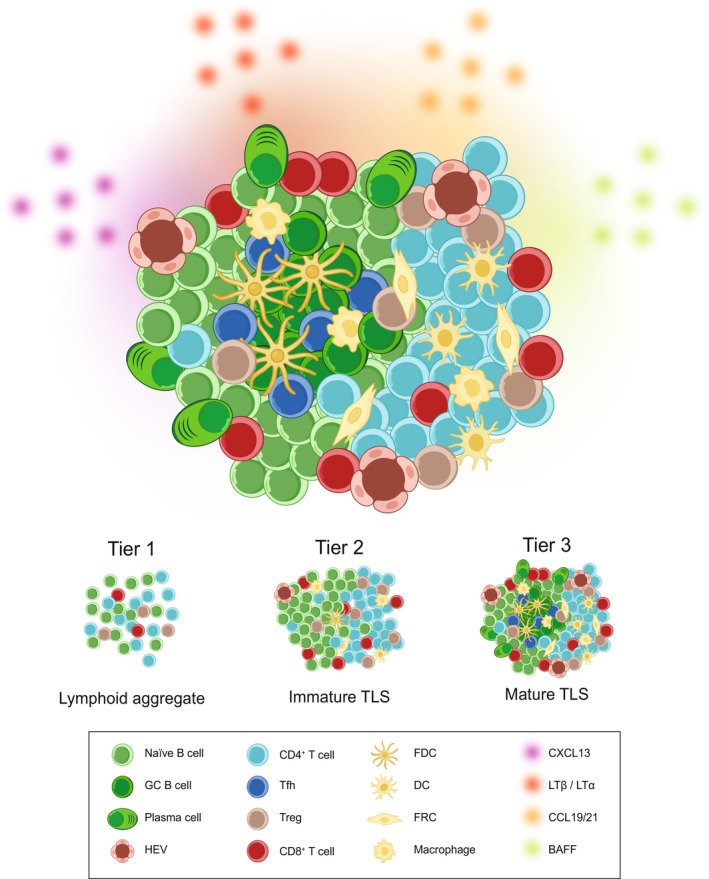
Cellular and Molecular Composition and Maturation States of TLSs. Main diagram: Schematic representation of key immune and stromal cell types commonly found in TLSs, including germinal center (GC) B cells, plasma cells, T follicular helper (Tfh) cells, regulatory T cells (Tregs), naïve B cells, CD4^+^ and CD8^+^ T cells, follicular dendritic cells (FDCs), dendritic cells (DCs), fibroblastic reticular cells (FRCs), macrophages, and high endothelial venules (HEVs), along with key signaling molecular mediators, including CXCL13, LTα/LTβ, CCL19/21, and BAFF. Bottom panel: Schematic representation of three proposed maturation tiers of TLSs, ranging from Tier 1 (lymphoid aggregates lacking clear zoning, FDCs, or HEVs) to Tier 2 (immature TLSs with partial organization, FDC and stromal networks, and early HEVs) and Tier 3 (GC‐containing mature TLSs).

The cellular composition of TLSs is remarkably diverse and may be influenced by several factors, such as the nature of the triggering antigen, tissue localization, and the stage of TLS maturation [1]. Despite this variability, certain components are consistently reported across tissues and disease contexts (Figure 1). Stromal cells, including fibroblasts, endothelial cells, and follicular dendritic cells (FDCs), play essential structural and functional roles, establishing the microenvironment that supports lymphoid organization and immune cell recruitment [2, 3]. B cell subsets within TLSs range from naïve and memory B cells to plasma cells (PCs) and often include GC B cells and, in some settings, regulatory B cells [1, 4, 5]. T cells are equally diverse, with TLSs frequently enriched in CD4^+^ and CD8^+^ subsets, including CD4^+^ T‐follicular helper cells (Tfh) in GC‐containing TLSs, as well as regulatory T cells, central memory, tissue‐resident memory, and exhausted T cells, reflecting the functional and temporal dynamics of the local immune response [6]. Myeloid cells such as conventional dendritic cells (DCs) and macrophages are also integral components. Conventional DCs facilitate antigen presentation and T cell activation through sustained cell–cell interactions [7]. Macrophages, which help clear apoptotic B cell clones in SLOs (as tingible body macrophages), have similarly important roles in TLSs within tissues like the gut and lung [8, 9]. Together, these populations orchestrate localized immune responses and underscore the complex immunological architecture of TLSs.

TLSs are not static entities but are thought to progress through distinct organizational states, from loosely associated lymphoid aggregates to fully developed GC‐containing structures [1, 5, 6, 10]. A commonly proposed framework groups these into three broad tiers. At the earliest stage (Tier 1), small perivascular clusters of B and T cells lack clear compartmentalization, FDCs or stromal FDC‐like cells, and high endothelial venules (HEVs). Tier 2, or immature TLSs, display more defined segregation of B cell‐ and T cell‐rich areas, often accompanied by FDC networks or stromal FDC‐like cells and early HEV formation; these structures can support antigen presentation and sustained T‐B cell interactions. Tier 3, or mature TLSs, are the most highly organized form, with T cell‐rich zones adjacent to B cell‐rich zones containing GCs. These GCs harbor proliferating Ki‐67^+^, activation‐induced cytidine deaminase (AID)^+^, and B‐cell lymphoma (BCL)‐6^+^ B cells, Tfh cells, and plasmablasts, and support affinity maturation, class switching, and somatic hypermutation to varying degrees [1, 11]. In cancer and other disease contexts, the presence of GC‐containing TLSs has been associated with improved immune responses and, in some settings, better clinical outcomes, reflecting their potential to enhance antigen presentation, cytokine‐mediated signaling, and antibody production [12, 13, 14, 15, 16]. Across all stages, TLSs may include diverse T cell subsets (naïve, effector, central memory, regulatory, dysfunctional, and exhausted) and B cell subsets (naïve, pre‐GC, GC, memory, plasma cells, and atypical). While this tiered framework can help standardize descriptions across studies, it remains a proposed construct rather than an established canon, and accurately determining TLS maturation status remains challenging due to the absence of a universally accepted classification system. The criteria used to define “mature” TLSs are not well standardized, and reports vary in the strength of supporting evidence, preventing a single classification scheme from being applied across all tissues and disease contexts. Nevertheless, advances in imaging, spatial transcriptomics, and multiplexed phenotyping are beginning to refine our understanding of TLS maturation, offering the potential for more precise and consistent classification in the near future [17, 18, 19, 20, 21].

Unlike GCs that form within SLOs after antigen exposure [11], ectopic GCs typically arise in non‐lymphoid tissues in response to chronic antigenic and inflammatory stimuli. Despite their different ontogeny, GC B cells in both settings undergo affinity maturation through somatic hypermutation of their B cell receptor (BCR) genes [11], altering their binding affinity for their cognate antigen. These acquired mutations are then evaluated in the light zone of the GCs by FDCs and CD4^+^ Tfh cells [11, 22, 23]. Over time, GC reactions generate broadly reactive memory B cells, capable of rapid reactivation upon re‐exposure, and high‐affinity long‐lived PCs that continuously produce protective antibodies [11, 24, 25].

Ectopic GCs have now been identified and characterized in a wide range of organs [26], including the lung [27, 28, 29, 30, 31], liver [32, 33, 34, 35, 36], brain meninges [37, 38, 39, 40, 41], skin [42, 43, 44], and blood vessels [45, 46, 47], among others. They arise not only in response to infection but also in various pathological contexts, such as autoimmune diseases (e.g., Wegener's granulomatosis [48], Hashimoto's thyroiditis [49], lupus [50, 51], rheumatoid arthritis [52, 53, 54], and primary biliary cholangitis [33]), organ transplant and graft rejections [55, 56], and multiple cancers [5, 6, 12, 14, 57, 58], including melanoma [14], non‐small‐cell lung cancers [59], hepatocellular carcinoma [60], and pancreatic cancer [61].

In this review, we outline current understandings and recent advances in TLS formation, structure, and function across diverse anatomical sites and disease settings. While shared features across TLS in several non‐lymphoid tissues have emerged, it remains unclear to what extent their development is shaped by local tissue environments, as it is rare for the same stimuli to induce TLS across multiple tissues simultaneously. We also explore the dual roles of TLS, either promoting protective immunity or driving pathology, depending on the context in which they arise. Given their presence in a wide range of conditions, TLSs have emerged as promising therapeutic targets: enhancing their formation may strengthen immune responses, while limiting their development could help control chronic inflammation or autoimmunity. By dissecting the mechanisms underlying TLS biology, we aim to highlight how these insights could inform novel therapeutic strategies across a spectrum of immune‐mediated diseases.

## 2 TLS At Different Anatomical Sites

**FIGURE 2:**
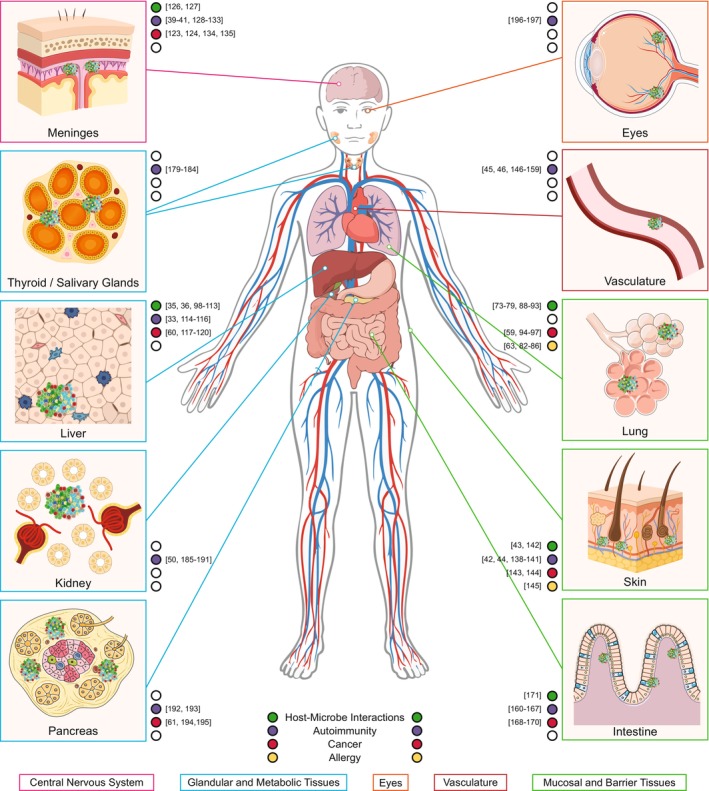
TLS heterogeneity across tissues and disease contexts. TLSs have been identified across a wide range of tissues, including the central nervous system, lung, skin, intestine, liver, kidney, pancreas, thyroid, salivary glands, vasculature, and eyes, where they contribute to immune responses in diverse pathological contexts such as infection, autoimmunity, cancer, and allergy. Organ‐ and context‐specific differences in TLS prevalence and structure are illustrated. The most relevant references for each disease and tissue type in which TLSs have been identified are indicated within the figure.

TLSs are not uniform entities but instead emerge within the unique physiological, anatomical, and immunological contexts of the tissues in which they form. Factors such as stroma and parenchymal composition, vascularization, metabolic tone, microbial exposure, and the identity of resident immune cells can profoundly shape TLS architecture, maturation, and function. As a result, TLSs display remarkable heterogeneity across organs, from the lung and liver to the brain and skin, each providing distinct microenvironmental cues that influence their development and dynamics (Figure 2). This diversity reveals a spectrum of inducible immunological niches, potentially fine‐tuned by local tissue signals. In the following sections, we detail the presence and features of TLSs across different tissues and disease settings, illustrating their spatial organization, cellular composition, and pathological or protective roles.

### 2.1 TLS in the Lung

As one of the largest barrier tissues to directly interact with the external environment, the lungs must be able to maintain robust immune protection while continuing to facilitate efficient gas exchange. The alveoli lack mucous, as this would impair gas exchange, which leaves the lower respiratory tract vulnerable to microbes that bypass the upper respiratory tract [62]. Instead, the lungs contain alveolar macrophages, which extend dendritic protrusions between epithelial cells to probe the luminal environment for foreign material [9, 63]. Viruses, bacteria, and fungi that enter the lower respiratory tract may infect the epithelium, which commences a signaling cascade leading to an inflammatory response and the beginning of an immune response in the lungs.

In the lung, three broad types of pulmonary lymphoid aggregates have been described: nodular inflammatory foci, which are sites of T cell priming [64]; granulomas, such as those typically associated with *Mycobacterium tuberculosis* infection [65]; and inducible bronchus‐associated lymphoid tissue (iBALT) [66]. While nodular inflammatory foci lack a B cell follicle and granulomas lack an intrinsic GC structural network, iBALT is composed of a T cell zone and B cell follicle, often also containing an active GC [67]. These iBALT can house structured aggregates of lymphocytes that resemble the form and function of canonical GCs [67]. Here we will focus on iBALT structures as the canonical pulmonary TLS.

**FIGURE 3:**
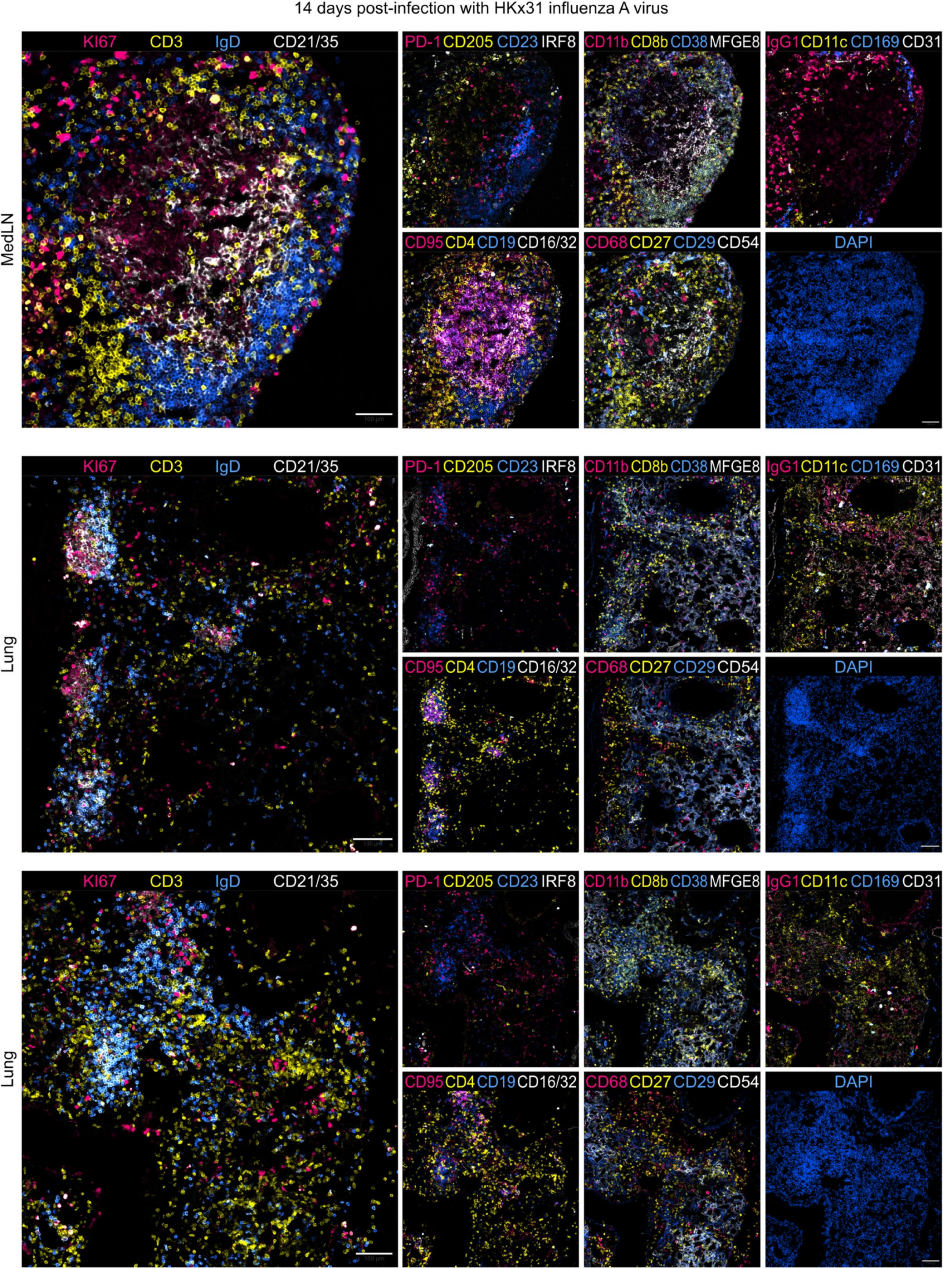
Intranasal HKx31 influenza A virus infection induces germinal center formation in the mediastinal lymph nodes and ectopically in the lung. Representative cyclical immunofluorescence images showing germinal centers in the mediastinal lymph node (top) and ectopically in the lung (center and bottom) following intranasal HKx31 influenza A virus infection. Images include staining for the indicated markers and DAPI. Scale bars, 100 μm. Tissue processing, image acquisition, and analysis were performed by Dr. Isabel San Martín Molina.

The lungs of murine neonates and human children are more permissive to iBALT formation than those of adults. In humans, iBALT structures are commonly observed during early childhood, with peak prevalence around three years of age, after which it gradually declines [67, 68, 69]. In adult mice and humans, iBALT typically forms in response to infection or chronic antigen stimulation [63, 70, 71]. Structurally, iBALT is usually located on the basal side of the bronchial epithelium, often adjacent to major blood vessels [72]. Lung inflammation is a potent inducer of ectopic GC formation, and iBALT can develop in response to a wide range of stimuli, including viral infections [73, 74, 75], such as respiratory syncytial virus, influenza (Figure 3), and coronaviruses, as well as bacterial pathogens like *Pseudomonas aeruginosa* [76], fungi such as *Pneumocystis jirovecii* [77], helminths like *Ascaris suum* [78], and environmental triggers including allergens [79] (e.g., house dust mite, pollen) and pollutants [63, 80] (e.g., cigarette smoke). T_H_2 and T_H_17 responses in the lung can mediate protection, as seen in iBALT formation during house dust mite exposure [27, 81]; however, repeated allergen exposure may drive excessive eosinophil recruitment and antibody production, contributing to the pathogenesis of asthma and chronic obstructive pulmonary disease [82, 83, 84, 85, 86].

**FIGURE 4:**
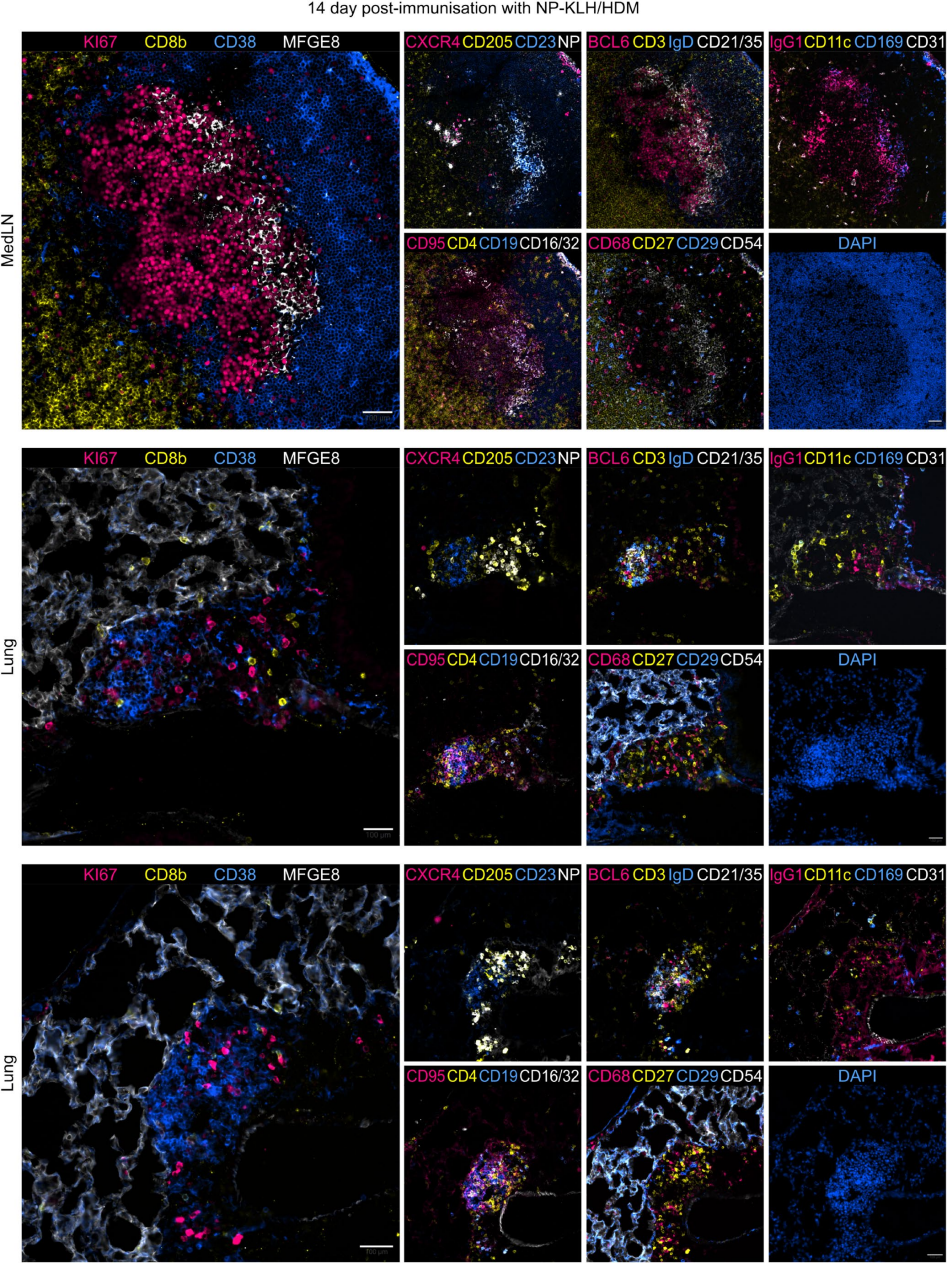
Intranasal NP‐KLH/HDM sensitization induces germinal centers in the mediastinal lymph node and ectopically in the lung. Representative cyclical immunofluorescence images showing germinal centers in the mediastinal lymph node (top) and ectopically in the lung (center and bottom) after intranasal NP‐KLH and house dust mite (HDM) sensitization. Images include staining for the indicated markers and DAPI. Scale bars, 100 μm. Tissue processing, image acquisition, and analysis were performed by Dr. Isabel San Martín Molina.

In the lung, epithelial and endothelial cells can stimulate local inflammation in response to infection or allergenic stimuli [87]. Activated stromal cell precursors in the lung differentiate into iBALT‐supporting stromal cells, including FDCs, HEVs, lymphatic endothelial cells, and structural fibroblasts [27]. Sustained local inflammation, together with chemokine production by macrophage and stromal cells, including CXCL12, CXCL13, CCL19, and CCL21, drives the recruitment of CXCR5^+^ B cells and T cells to the developing niche. This coordinated process promotes maturation of distinct follicular and T cell zones, ultimately giving rise to GC structures [88]. Structurally, ectopic GCs in the lung share many key features with conventional GCs in SLOs, including the expression of conventional GC B cell markers such as AID, GL‐7, BCL‐6, CD95, and Ki‐67, and exhibit polarization into dark and light zones [74, 75, 79, 89]. Allergen‐induced lung GCs exhibit this functional compartmentalization (Figure 4) and are functionally competent, as they support affinity maturation in a similar way to conventional GCs in LNs, although with delayed kinetics [75, 79]. These structures are also capable of locally generating lung memory B cells, independent of those derived from the lung‐draining LNs [79, 90], a population shown to be essential for recall responses during reinfection [74, 91]. Studies in splenectomized LTα knockout mice (where most LNs fail to develop) have shown that iBALT can protect mice from infection, reinforcing their role as frontline defense against respiratory diseases [92]. Similar GC‐like structures have also been described in the nasal‐associated lymphoid tissue (NALT), forming independently of draining LNs, particularly in mice infected with influenza A virus [93].

Ectopic GCs in lung cancers are linked to patient outcomes; TLS development in proximity to non‐small‐cell lung carcinoma (NSCLC) has been associated with positive prognoses and greater patient survival [59, 94, 95, 96]. This beneficial antitumoral response has been attributed to increased local frequencies of DCs and T cells, where the density of mature DC‐LAMP^+^ DCs, FDCs, T cells, and B cells correlated with each other and with favorable outcomes to antitumor therapies [59, 95, 96]. Notably, low‐dose radiotherapy in NSCLC patients has been shown to enhance both the number and maturity of iBALT structures, thereby improving responses to α‐PD‐1 treatment and extending survival [97]. These findings underscore the potential of TLSs to support effective antitumor immunity and highlight the need for further research into how their formation and function can be therapeutically modulated. Collectively, these observations illustrate the dual nature of lung TLSs in mediating either protective or pathological outcomes, depending on the context. Continued investigation is essential to delineate the specific role of iBALT in respiratory disease and to harness TLS biology for therapeutic benefit.

### 2.2 TLS in the Liver

TLSs have emerged as dynamic immunological niches in the liver, observed across a range of pathological conditions. Infections, both viral and bacterial, have been linked with TLS development. In hepatitis C virus (HCV) infection, sustained hepatic inflammation drives the formation of TLS‐like aggregates, often located in the portal tracts and interlobular bile ducts [98, 99, 100, 101]. These structures typically consist of a B cell‐rich core with interspersed T helper cells, surrounded by cytotoxic/suppressor T cells [102], and frequently exhibit B cell receptor clonality indicative of independent and localized B cell expansion [35, 103, 104]. Mature TLSs with GC features have also been described, showing functional parallels to SLOs, including the expression of Ig isotypes and GC markers such as Ki‐67, CD23, BCL‐2, and BCL‐6 [36, 105]. In hepatitis B virus (HBV) infection, B cells are essential for antiviral defense [106, 107], yet become functionally impaired during chronic infection, often adopting atypical memory phenotypes and exhibiting a reduced capacity to differentiate into antibody‐secreting cells [108, 109]. Although TLSs are less frequently reported with HBV than with HCV, they have been identified in the periportal areas of chronic HBV livers, where lymphoid aggregates are composed of T cells, B cells, and macrophages [110]. Similar findings have been reported in HBV‐infected chimpanzees treated with the toll‐like receptor (TLR) 7 agonist GS‐9620, which triggered intrahepatic interferon responses and TLS formation [111]. Despite these advances, the formation of TLS in HBV infection, particularly outside the context of chronic disease, remains poorly understood, in part due to the scarcity of suitable experimental animal models. Emerging evidence shows that HBV‐replication‐competent transgenic mice can host CD4^+^‐dependent TLSs in periportal regions, displaying GC‐like features, including GC B cells, Tfh cells, local antibody production, and PC differentiation. TLR signaling was also shown to induce intrahepatic myeloid cell aggregates that promote TNF‐dependent expansion of cytotoxic T lymphocytes, predominantly during acute viral infection [112]. Beyond viral infections, bacterial infections also reveal contrasting TLS dynamics: in *Helicobacter pylori* ‐infected mice, TLSs emerged in inflamed liver regions, featuring B and T cells, MAdCAM‐1^+^ PNAd^+^ HEVs, and expression of *Ccl21* and *Cxcl13* [34], whereas *Ehrlichia muris* infection failed to induce TLSs or GCs despite evidence of somatic hypermutation and class switching in liver‐resident B cells [113].

TLSs are also a hallmark of chronic autoimmune liver diseases. In conditions such as primary biliary cholangitis and primary sclerosing cholangitis, lymphoid aggregates often form in portal tracts near damaged bile ducts. These structures contain organized B and T cell zones, DCs, and HEV‐like vasculature expressing CD34 and MAdCAM‐1 [114, 115]. The strong expression of CCL21 within these aggregates likely supports lymphocyte recruitment and retention. While B cells were traditionally viewed as antigen‐presenting cells in this context, recent findings suggest that CD38^+^ PCs may play a more direct pathogenic role in bile duct injury, as their coronal arrangement around bile ducts is linked to primary biliary cholangitis pathogenesis [116]. TLS maturation in primary biliary cholangitis has been linked to poor prognosis, with fully formed GCs containing CD20^+^ B cells and CD21^+^ FDCs surrounded by CD4^+^ and CD8^+^ T cells and MUM1^+^ PCs. Importantly, both CD8^+^ T cells and PCs were observed infiltrating the biliary epithelium, suggesting direct cytotoxic effects [33]. Similar TLSs are present in autoimmune hepatitis, though less frequently and often with less well‐defined organization. Lymphocytes in autoimmune hepatitis tend to spread from portal areas into lobular regions, forming a more diffuse interface rather than discrete follicles [33].

In the context of cancer, TLSs exhibit complex and context‐dependent roles in the liver. In hepatocellular carcinoma, TLSs have been associated with both pro‐tumor and antitumor effects. Some studies describe them as immunopathological “microniches” that support the expansion of malignant progenitor cells within a cytokine‐rich environment [117]. Conversely, the presence of intratumoral TLSs has been linked to reduced early recurrence and improved survival following surgical resection [118], with TLS‐associated gene signatures proposed as potential prognostic biomarkers [119]. Recent work by Shu et al. revealed that neoadjuvant immune checkpoint blockade therapy enhances TLS formation within tumors, where mature TLSs contain AID^+^ BCL‐6^+^ B cells and T cells arranged in GC‐like structures. Notably, even regressing tumor regions retained “involuted” TLSs enriched with effector and tissue‐resident memory CD8^+^ T cells, suggesting a continued role in immune surveillance after tumor reduction [60]. TLS involvement in antitumor immunity is not limited to hepatocellular carcinoma. In APC‐mutated hepatoblastoma, a pediatric liver tumor, cisplatin treatment induced intratumoral TLSs with dense CD3^+^ T cell infiltrates, sparse CD20^+^ B cells, and absent GCs, though the presence of DC‐LAMP^+^ dendritic cells suggested ongoing antigen presentation [120].

Finally, aging represents a non‐pathogenic but biologically significant context in which TLSs arise in the liver. In 24‐month‐old mouse livers, perivascular immune aggregates display defining features of TLSs, including proliferating T and B cells, DCs, and elevated expression of chemokines such as RANTES, MIP‐1α, CXCL1, and CXCL13, along with increased LTα and PNAd [32]. In a separate study using 24‐month‐old mice, these TLSs were found to form spontaneously in the absence of overt liver injury and contain senescence‐associated T cells (CD45^+^ TNFSF8^+^ CD3ε^+^) and age‐associated B cells (CD45^+^ TNFRSF8^+^ CD19^+^). These findings are echoed in human studies, where older HCV‐infected patients exhibited higher expression of TLS‐related genes and a 100% prevalence of hepatic TLSs in liver biopsies [121]. Together, these observations suggest that TLS formation may represent a conserved immunological response to chronic stimulation, even in the absence of classical disease.

### 2.3 TLS in the Central Nervous System (CNS)

TLS has been identified across various CNS conditions, including infections, autoimmunity, and gliomas [38, 122]. Unlike their counterparts in non‐CNS tissues, CNS‐associated TLS display distinct structural and spatial characteristics. Rather than infiltrating the brain parenchyma, they primarily localize near meningeal tissues, such as the cortex‐associated meninges and the choroid plexus [123, 124]. This peripheral positioning, particularly along the cortical surface, often leads to an elongated morphology uncommon in other tissues. The preference for meningeal localization also reflects the specialized stromal cell architecture and immune‐regulating barriers unique to the CNS. While stromal cells like fibroblasts and lymphatic endothelial cells are widely distributed within the parenchyma of peripheral organs, their presence in the CNS is largely restricted to meningeal compartments [125].

Pathogens like *Mycobacterium tuberculosis* [126] and *Trypanosoma brucei* [127] can drive TLS formation in the meninges, often resembling structured SLOs that facilitate granuloma formation, infection containment, and GC‐like immune responses. The case of chronic *Trypanosoma brucei* infection is particularly striking, as TLS acquire features typical of ectopic GCs, with structures containing FDC‐like cells, Tfh cells, and class‐switched B cells [127]. However, these responses may come at a cost; autoreactive B cells producing IgG against both parasitic and host antigens, such as myelin basic protein, suggest a heightened risk of infection‐induced autoimmunity within the CNS.

In autoimmune conditions such as multiple sclerosis [41] and experimental autoimmune encephalomyelitis [39], TLSs containing ectopic GCs are frequently detected within the meninges of the cerebral hemispheres, particularly in regions of the cerebellum, brainstem, spinal cord, and deep sulci [40, 41, 128, 129]. Their presence is often linked to advanced and aggressive disease stages, with GC‐like structures associated with increased cortical demyelination, inflammatory infiltration, and accelerated disease progression [40, 41]. Recent findings demonstrate that T and B cells form long‐lasting, antigen‐specific contacts within these meningeal TLSs, promoting both B cell differentiation and reactivation of autoreactive T cells, thereby sustaining pathogenic inflammation in the CNS [130]. In experimental autoimmune encephalitis, disease progression has also been associated with the expression of BAFF, a B cell survival protein, and the chemokine CXCL13 [131]. The detection of these structures is inconsistent across TLSs, reflecting variability in their formation and functional capacity. In multiple sclerosis, meningeal TLSs contain B cells, T cells, PCs, and macrophages, but often lack HEVs [41]. While B cell‐depleting therapies such as α‐CD20 effectively reduce circulating B cells, they often fail to eliminate meningeal TLSs, suggesting their persistence is independent of peripheral B cell availability [132]. Although α‐CD20 therapies fail to eliminate meningeal TLS, Siponimod, which promotes retention of B and T cells inside LNs, effectively reduces TLS growth in experimental autoimmune encephalomyelitis models [133], a widely used mouse model of multiple sclerosis that recapitulates key features of CNS autoimmunity, including demyelination, immune cell infiltration, and neurological deficits.

TLSs are commonly detected in untreated human gliomas [123, 134] and in orthotopic mouse glioma models using GL261 or CT‐2A cells injected into the brain [123], where their presence is generally associated with better prognosis and enhanced responses to immunotherapy. Interventions promoting CD40 signaling, such as agonistic CD40 antibodies and adenovirus‐encoded CD40L, effectively induce TLS formation by driving B cell aggregation near meningeal tissues and within the brainstem [135]. Additionally, the administration of an adeno‐associated virus encoding LIGHT, which targets brain endothelial cells, fosters the formation of tumor‐associated HEVs and T cell‐rich TLS, which enhance antitumor immunity and prolong survival in α‐PD‐1‐resistant glioma models [124]. Overall, CNS‐associated TLSs are highly context‐dependent structures whose meningeal localization and unique microenvironment shape both protective and pathogenic outcomes. While they can contribute to antitumor immunity and infection control, their involvement in autoimmune diseases poses significant challenges. Nevertheless, emerging therapies that target pathogenic TLS formation offer promising avenues to modulate CNS inflammation and improve patient outcomes.

### 2.4 TLS in the Skin

Although TLSs have been well‐documented in other organs, their presence in the skin has only recently been reported and remains comparatively less understood. Emerging evidence indicates that skin‐associated TLSs can form in response to diverse stimuli, including infectious agents, chronic inflammatory conditions, autoimmune diseases, cancer, and allergic inflammation [4, 136, 137]. Notably, their formation within the skin appears to be influenced by unique local factors, such as commensal bacteria and tissue‐specific immune cell repertoires, which are likely to shape both their development and function in this distinct environment [43].

In inflammatory skin diseases such as hidradenitis suppurativa [42], pyoderma gangrenosum, and pemphigus [44, 138], high numbers of B cells and PCs accumulate within lesions. Hidradenitis suppurativa lesions, for example, contain a mixture of naive, activated, and class‐switched memory B cells, along with PCs [42]. Both TLSs and autoantibodies have been identified within these lesions, suggesting a tissue‐intrinsic mechanism for B cell differentiation that may contribute to disease pathology. TLSs are commonly found in pemphigus vulgaris and foliaceus lesions, with the presence of desmoglein‐specific B cells, CD138^+^ PCs, HEVs, and activated CD4^+^ T cells, predominantly T_H_1‐like cells producing CXCL13 [44, 138]. Lymphoid follicles are also detected in skin lesions of cutaneous lupus erythematosus [139, 140], and psoriasis [141]. Psoriatic lesions can contain HEVs and clusters of DCs and T cells expressing CCL19 and CCL20, which contribute to inflammation maintenance [141].

Recently, TLSs have been identified in skin colonized by *Staphylococcus epidermidis* , featuring GCs that sustain local antibody production and provide systemic protection [43]. Their BCR repertoires differ from those in lymph nodes, suggesting that unique antigenic encounters in the skin contribute to their diversification. The development and maintenance of these structures is critically dependent on Tfh cells, which in the skin can arise from regulatory T cells that lose FoxP3 expression [43]. TLS‐like structures have also been reported in the skin lesions of secondary syphilis. These lymphoid clusters contain CXCL13^+^ fibroblast‐like cells in association with B cells and Tfh cells, suggesting active immune responses at these sites [142].

TLSs have also been identified in skin cancers, particularly melanoma, where they are associated with favorable clinical outcomes [143]. These structures often feature HEVs, B cells, T cells, and mature DCs [144]. In allergic conditions such as allergic contact dermatitis, DC‐T cell clusters in the dermis are associated with inflammatory cytokine production [145].

### 2.5 TLS in the Vasculature

TLS formation around blood vessels is a feature of various inflammatory conditions, including vasculitis and atherosclerosis [45, 46]. In autoimmune vasculitis, TLSs frequently develop around adventitial vasa vasorum, where they sustain chronic inflammation and promote tissue damage. Within these structures, a distinct population of TCF‐1^+^ IL‐7R^+^ CD4^+^ T cells with stem cell‐like properties resides within these structures, providing a continuous supply of effector T cells that drive disease pathology. These progenitor‐like cells exhibit high proliferative capacity and differentiate into Eomes^+^ cytotoxic T cells and BCL‐6^+^ Tfh cells, actively contributing to inflammation and disease progression [45]. In giant cell arteritis, TLSs, often containing GCs and closely associated with HEVs, are commonly identified within aortic and temporal artery tissues where molecular signals such as CXCL13, BAFF, APRIL, LTβ, IL‐17, and IL‐7 drive their formation and maintenance, thereby perpetuating chronic inflammation and promoting autoimmune tissue damage [46].

Earlier observations indicate that atherosclerotic lesions in humans can also harbor both TLS‐like structures [146] and antibody deposits [147, 148, 149, 150], suggesting a site‐specific immune response. In fact, these artery tertiary lymphoid organs form within the lamina adventitia of large and medium‐sized arteries, where they develop a compartmentalized organization of T cell zones, B cell follicles, and specialized HEVs [47, 151, 152, 153, 154]. Immunohistochemical analyses have revealed that these aggregates also contain fibroblastic reticular cells, FDCs, macrophages, and proliferating lymphocytes, as well as class switching and local antibody production [147, 155, 156]. Situated adjacent to atherosclerotic plaques, artery tertiary lymphoid organs are driven by signals such as CCL21 and CXCL13 secreted by medial vascular smooth muscle cells [154, 157]. Among the notable immunogenic targets in advanced plaques are stress‐induced mitochondrial antigens, including ALDH4A1, where infusions of α‐ALDH4A1 antibody have been shown to lower lipid levels and slow disease progression, underscoring the therapeutic significance of these localized immune responses [158, 159].

### 2.6 TLS in the Intestine

TLS formation in the gut is orchestrated by interactions among neural, immune, and stromal elements, and they are evident in models of colitis, Crohn's disease, and colorectal cancer. In colitis lesions, intense inflammation is associated with abundant TLSs, where CXCL13 and its receptor CXCR5, normally present in gut‐associated lymphoid tissue, also promote the formation of aberrant aggregates in ulcerative colitis [160]. Experimental models further reveal that activation of the vagus nerve stimulates intestinal neurons to induce CXCL13 production in stromal cells, a mechanism that is lost following vagotomy, even though colitis itself continues to progress [161]. Mechanistic insights have been obtained from studies in RORγt‐deficient mice. Even in the absence of traditional lymphoid tissue inducer cells like T_H_17 cells, TLSs can be induced, relying on B cell‐expressed LTβ. However, this compensatory formation of TLSs in the face of an uncontrolled microbiota can exacerbate inflammatory pathology [162]. In Crohn's disease, ectopic TLSs were also found at the base of aphthous ulcers and are associated with increased local production of CCL19 and CCL21. This shift in chemokine gradients causes infiltrating CCR7^+^ T cells to be retained, contributing to the perpetuation of chronic ileitis [163, 164, 165]. Moreover, inflammatory mediators like TNF‐α and LPS can activate perivisceral adipocytes to serve as organizers for TLS formation, a process linked to more severe disease phenotypes [166, 167].

Beyond inflammatory bowel diseases, TLSs have significant prognostic value in colorectal cancer. Intratumoral TLS enriched in Tfh cells correlates with improved survival, whereas peritumoral TLS often predicts poorer relapse‐free and overall survival [168]. Moreover, mature TLS with abundant HEVs enhances the recruitment of CD3^+^ and CD8^+^ T cells and macrophages, associating with better overall and disease‐free survival and lower tumor staging [169, 170]. Complementing these findings, *Helicobacter hepaticus* colonization in a mouse model of colorectal cancer can enhance the maturation of tumor‐adjacent TLSs, thereby promoting antitumor immunity through the coordinated action of *Helicobacter hepaticus* ‐specific Tfh cells, B cells, and NK cells [171].

### 2.7 TLS in Additional Organs: Thyroids, Salivary Glands, Kidney, Pancreas and Eyes

#### 2.7.1 Thyroid

In the early 1970s, Söderström et al. observed that the thyroid gland in Hashimoto's thyroiditis exhibits a lymph node‐like architecture [172, 173], a feature now attributed to the near‐ubiquitous presence of TLSs in this disease [49]. It was documented that these TLS, which frequently contain ectopic GCs, arise from intricate chemokine and cytokine networks: for instance, the CCL21‐CCR7 axis draws T cells and CD11c^+^ dendritic cells to the thyroid, with LTβ signaling further supporting TLS formation [174]. Autoimmune thyroid glands with ectopic GCs exhibit elevated levels of LTα, IFN‐γ, CXCL12, CXCL13, and CCL22, while the thyroid epithelium itself can secrete CXCL12 under inflammatory stress, thereby fostering local autoantibody production [175]. LTβR‐mediated pathways further contribute to inflammation [176], and transgenic mice expressing CCL21 under the thyroglobulin promoter develop extensive lymphocytic infiltrates and HEVs within the thyroid, underscoring the key role of CCL21 in autoimmune thyroid disease [177]. More recently, a stromal subset of myofibroblasts partially surrounding ACKR1^+^ HEVs was shown to promote TLS formation in Hashimoto's thyroiditis, with abundant GC B cells and PCs indicating that the thyroid is a major source of autoimmune antibodies [178].

#### 2.7.2 Salivary Glands

In Sjögren's syndrome (SS), a chronic autoimmune disease that primarily targets exocrine glands, the salivary glands are a key site of TLS formation. A defining feature of SS is the presence of TLSs with GCs, which function as local sites of autoantibody production [179, 180]. These ectopic lymphoid aggregates sustain T–B cell interactions, promoting the clonal expansion of autoreactive B cells and amplifying local inflammation [181]. Within these TLSs, immune activation is further marked by upregulation of NCR3 (NKp30) and effector molecules such as granzyme B, perforin, and IFN‐γ, indicating heightened cytotoxic activity. Importantly, rituximab treatment has been shown to prevent NKp30 upregulation in the salivary glands while also disrupting B cells, FDC networks, and GC structures, thus destabilizing TLS architecture [182]. Additional studies have shown that elevated levels of pro‐inflammatory cytokines and chemokines, including type I interferons, further contribute to the development and maintenance of TLSs [183, 184]. Over time, chronic TLS activity sustains the production of autoantibodies, most commonly targeting Ro/SSA and La/SSB antigens, leading to progressive glandular dysfunction and local tissue damage [179].

#### 2.7.3 Kidney

Systemic lupus erythematosus is a chronic autoimmune disease with lupus nephritis as one of its most severe organ manifestations, characterized by immune complex deposition and renal inflammation [185]. Under these chronic inflammatory conditions, mesenchymal stem cells can act as tissue organizers in the formation of kidney‐specific TLSs, thereby accelerating disease progression [50]. Studies show that CXCL13‐dependent TLS presence correlates with a longer disease course and reduced therapy responsiveness [186]. In micro‐dissected TLSs from patient kidney biopsies, clonal B cells with a high degree of somatic hypermutation were identified, indicating in situ GC reactions locally contribute to autoantibody production and further renal damage [51, 187]. The presence of Tfh cells was correlated with worse renal function [188], underscoring the pathogenic potential of TLSs [189]. Beyond lupus nephritis, TLS development can drive the progression of kidney diseases such as acute kidney injury in the elderly [55, 190]. Age‐dependent TLS formation in the kidney appears conserved across species, with similar mechanisms seen in aged mice and elderly humans; indeed, these structures occur not only in immune‐mediated pathologies but also in diabetic nephropathy and benign nephrosclerosis, suggesting that TLS formation may be a universal feature of renal aging [191].

#### 2.7.4 Pancreas

TLSs in the pancreas can exert different effects depending on the underlying pathology. In a study involving type 1 diabetes patients, more than half of those presenting with insulitis developed TLSs; however, TLS frequency was found to decline as β cell loss progressed [192]. Moreover, studies in the non‐obese diabetic mouse model suggest that islet destruction may stem from compromised TLS integrity [193]. By contrast, in the highly lethal pancreatic ductal adenocarcinoma (PDAC), the presence of TLSs is associated with better patient outcomes and longer survival [61]. TLS‐enriched PDAC tumors harbor memory lymphocytes, IgG‐switched B cells, and robust T cell responses [15]. A mature TLS gene signature in pretreatment biopsies predicts improved survival following chemoimmunotherapy, while TLS positivity in surgically treated patients correlates with heightened infiltration of CD4^+^ and CD8^+^ T cells, B cells, and activated immune cells [194, 195]. Collectively, these findings underscore the pivotal role TLSs play in shaping both autoimmune and neoplastic processes in the pancreas.

#### 2.7.5 Eyes

TLSs have also been detected in the retinas of humans and mice with uveitis, suggesting a complex, localized immune response. Human studies further indicate that TLSs appear in roughly 20% of chronic, persistent uveitis cases, highlighting notable immune dysregulation in this disease [196, 197]. In a spontaneous autoimmune mouse model, retinal TLSs featured well‐organized B and T cell zones expressing GC markers, including PNA, GL‐7, and CXCR5, and harboring CD138^+^/B220^+^ PCs, implying that these structures may actively contribute to antibody production and disease progression [198].

Recent studies have demonstrated that TLSs can form in nearly all organs, from barrier sites like the lung and skin to immune‐privileged areas such as the CNS and eye, as well as deep tissues like the liver, pancreas, and kidney. Yet, their formation, organization, architecture, and function vary widely depending on the local tissue environment. Factors such as stromal architecture, vascular structure, antigen exposure, and resident immune populations shape distinct TLS phenotypes, leading to considerable heterogeneity across anatomical sites and disease settings. While TLSs may promote protective immunity in some contexts and sustain chronic inflammation in others, their impact appears tightly linked to the microenvironment in which they arise. However, a holistic review of the literature may reveal common features and shared factors that lead to TLS formation and function in many, if not all, tissues.

### 2.8 Common Features Mediating TLS Formation Across Organs

Despite the pronounced heterogeneity of TLSs across tissues, a cross‐organ analysis of the literature reveals that they do share core structural and molecular features. These include the presence of organized B and T cell zones, the establishment of chemokine gradients, most notably involving CXCL13, CCL19, and CCL21, stromal cell remodeling, and the local activation of antigen‐presenting cells. These conserved elements point to shared molecular programs underlying TLS induction, even as their structure and function are modulated by the unique microenvironment of each organ.

This convergence is striking, given the diversity of pathological triggers and anatomical niches in which TLSs form. However, systematic, multi‐organ comparisons remain scarce, largely due to the lack of models capable of inducing TLSs in multiple tissues simultaneously. As a result, much of what we know about TLS biology comes from isolated studies of individual tissues, making comparative conclusions challenging. Still, several key mediators have emerged as common drivers across systems. Among these, CXCL13 stands out as the most consistently associated with TLS formation. Its expression has been linked to TLS formation in a wide range of tissues and disease contexts: in the lung during influenza A virus infection [75], in the liver in response to *Helicobacter pylori* infection [34], in the CNS during experimental autoimmune encephalomyelitis [131], in the skin in pemphigus vulgaris and foliaceus lesions [44, 138], as well as following *Staphylococcus epidermidis* colonization [43], in the vasculature in giant cell arteritis and atherosclerotic plaques [46, 157], in the gut in ulcerative colitis [160], in thyroid glands in autoimmune thyroid disease [175], and in the kidneys in lupus nephritis [186]. Although CXCL13 is not universally required for TLS induction, it appears to play a central role in initiating TLS formation through recruitment and positioning of CXCR5^+^ B cells.

Other molecules, including LTβ and BAFF, also play recurring roles in TLS development, acting on stromal and immune compartments to promote lymphoid neogenesis. Systemic modulation of TLSs through strategies such as CXCL13 blockade, or conversely, CXCL13‐encoding mRNA or viral vectors, holds promise for either suppressing pathogenic TLSs or enhancing their formation in cancer immunotherapy settings [199]. although further research is required. However, more work is needed to determine whether these strategies can be fine‐tuned to specific tissues and disease states without unwanted systemic effects.

Beyond molecular mediators, the function and impact of TLSs are profoundly shaped by their anatomical and pathological context. As described above, TLSs have been identified in numerous tissues, where they adopt distinct roles depending on the local environment. In infections, they can act as frontline defense structures, supporting robust antigen‐specific responses and facilitating local memory formation [1, 26]. In cancer [6], TLSs embedded within tumors often correlate with enhanced immune surveillance, improved prognosis, and greater responsiveness to immunotherapy, as demonstrated in melanoma [143], hepatocellular carcinoma [119], pancreatic cancer [15], and gliomas [134]. Conversely, in autoimmune diseases [200] such as rheumatoid arthritis [52, 54], Hashimoto's thyroiditis [49], Sjögren's syndrome [179, 180, 201], and lupus nephritis [50, 51], TLSs may act as engines of chronic inflammation, driving autoantibody production and sustaining tissue damage. They have also been implicated in amplifying hypersensitivity reactions in allergic conditions [70, 83, 86]. Notably, TLS formation is not restricted to overt disease. These structures can also emerge during tissue repair, aging, and sterile injury, sometimes contributing to regeneration, but often promoting chronic inflammation and fibrosis [32, 55, 202, 203, 204].

Together, these findings underscore that while TLSs are shaped by their microenvironment, they are also guided by a set of common molecular and cellular mechanisms. This dual nature, context‐specific yet mechanistically conserved, offers both challenges and opportunities for TLS‐targeted therapies [1, 6]. Understanding which features are universal and which are tissue‐dependent will be key to developing interventions that harness the protective aspects of TLSs while minimizing their pathological consequences.

## 3 Clinical Relevance and Strategic Modulation of TLSs

TLSs exemplify the remarkable flexibility of the immune system to form lymphoid aggregates across a wide range of clinical contexts and tissues. Whether arising in response to infection to provide local protection, fueling chronic inflammation in autoimmunity, enhancing antitumor responses, or amplifying allergic reactions, TLSs span a full spectrum from protective to pathogenic. While often linked to prolonged antigenic stimulation, they can also emerge during acute inflammation, tissue remodeling, aging, or sterile injury, reflecting the interplay between local tissue cues and systemic immune demands. Far from being static or uniform, TLSs dynamically adapt their architecture and function to their tissue environment, underscoring their dual nature as both key protectors and drivers of disease.

**FIGURE 5:**
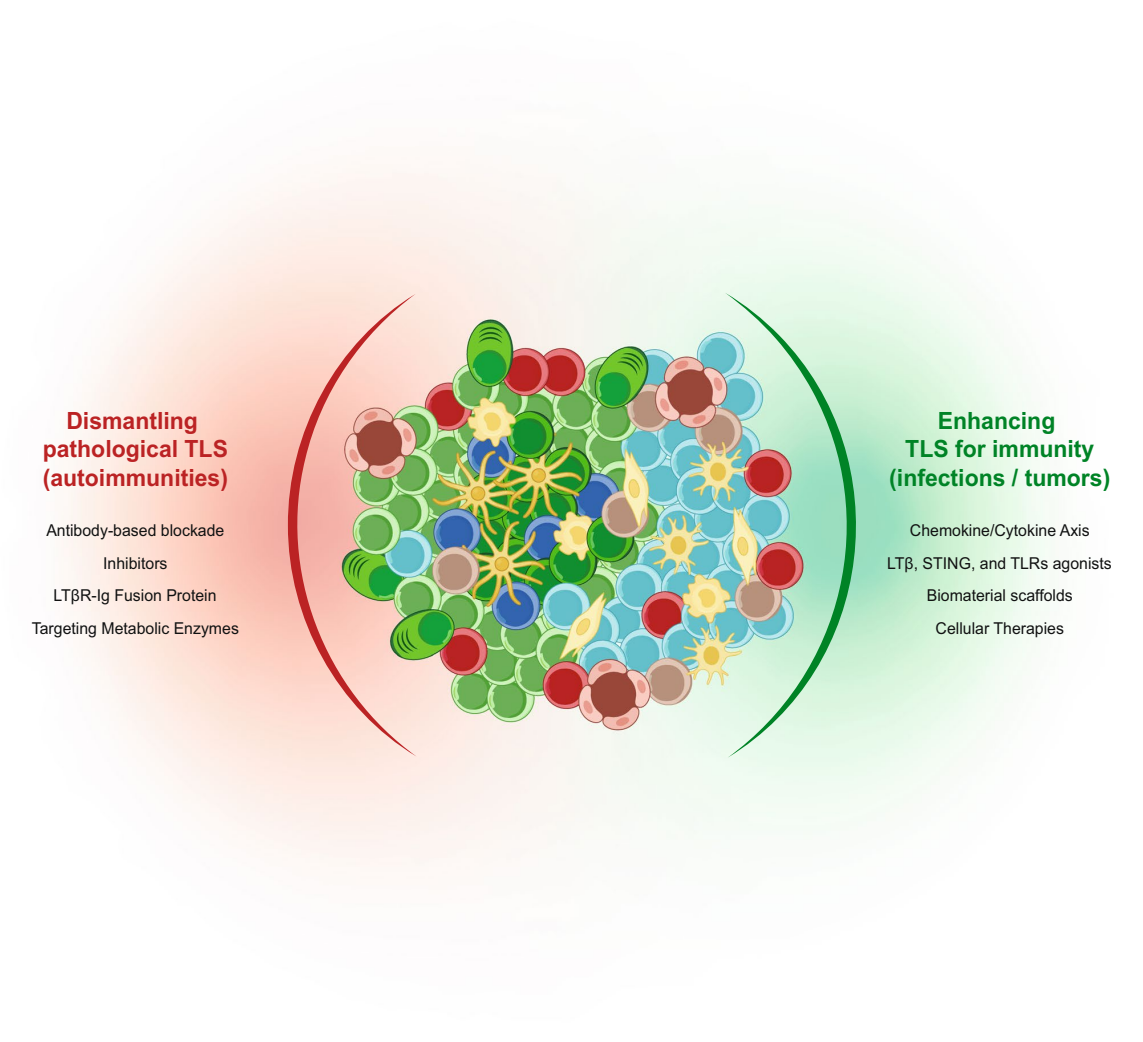
Therapeutic strategies to modulate TLS induction and function. Strategies to enhance TLS formation in the context of infections and tumors (left), including cytokine/chemokine delivery, TLR or STING agonists, biomaterial scaffolds, and cellular therapies. On the right are approaches to dismantle or suppress pathogenic TLSs in autoimmune diseases, including antibody‐based blockade, LTβR‐Ig fusion proteins, metabolic enzyme targeting, and chemokine inhibition.

This intricate balance between protection and pathology highlights the therapeutic potential and complexity of targeting TLSs. The following sections provide an in‐depth look at current approaches aimed at modulating TLSs, from molecular and cellular strategies to biomaterials and pharmacological interventions, as illustrated in Figure 5. Whether the goal is to establish robust local immune niches for therapeutic benefit or dismantle pathogenic ectopic lymphoid structures, a nuanced understanding of TLS biology is key to developing effective and precise therapies.

### 3.1 Inducing and Enhancing TLS Formation

Therapeutic induction or enhancement of protective TLSs represents a promising strategy for boosting local immunity in contexts like chronic infection or cancer immunotherapy. Cytokine and chemokine signaling play pivotal roles in initiating TLS formation, particularly through the recruitment of lymphocytes to non‐lymphoid tissues. Chemokines such as CXCL13 and CCL21 have been successfully used to drive TLS development in preclinical tumor models. For instance, recombinant CXCL13 promoted TLS formation and CD8^+^ T cell infiltration in ovarian cancer [205, 206], and co‐injection of CXCL13 and CCL21 with gemcitabine significantly enhanced TLS formation in pancreatic cancer models [207]. LIGHT has also emerged as a potent driver of vascular normalization and lymphoid neogenesis. Delivery via plasmid vectors, adeno‐associated viral vectors, or modified chimeric antigen receptor T cells has led to HEV formation and T cell infiltration in glioblastoma, glioma, and melanoma models, supporting TLS‐compatible niches [124, 207, 208, 209].

Beyond chemokines and cytokines, agonists targeting key immunoregulatory pathways such as LTβR, STING, and TLRs have shown promise in enhancing TLS features. LTβR activation with monoclonal antibodies strengthens fibroblastic reticular cell networks and increases HEV density and T cell infiltration in colon and pancreatic tumors [210, 211]. STING agonists like cGAMP and ADU‐S100 promote vascular remodeling, upregulate TLS‐related chemokines (LTα, LTβ, CCL19, CXCL13), and enhance infiltration by DCs and CD8^+^ T cells [212, 213]. While B cell recruitment is limited with STING activation alone, co‐administration with IFN‐β or additional adjuvants has demonstrated GC induction in mouse lungs after intranasal installation [75]. TLR4 and TLR7 agonists, including LPS and GS‐9620, have also been implicated in TLS neogenesis by enhancing CXCL13 expression and lymphoid aggregation in cancer and viral hepatitis models [111, 214].

Biomaterials and scaffold technologies have been used as physical frameworks and controlled‐release systems to foster TLS induction. Hydrogels incorporating CXCL13, CCL21, and LTα1β2 have successfully generated TLS‐like structures in murine kidneys and tumors, complete with lymphatic capillaries and stromal networks [215]. Advanced formulations such as ZCCG hydrogels, combining STING activation and CpG delivery, further stimulate DC maturation and cytokine production, creating pro‐inflammatory environments conducive to TLS formation [216]. Similarly, nanoscale peptide hydrogels (e.g., MRM‐coated spores) boost CXCL9 expression and CD8^+^ T cell responses, though their impact on TLS architecture remains under evaluation [217]. Synthetic scaffolds, including 3D‐printed constructs and polyurethane‐collagen matrices, have replicated features of secondary lymphoid organs, enabling lymphocyte recruitment, antigen‐presenting cell activation, and TLS‐like patterning [218, 219, 220, 221].

Cellular therapies represent an emerging class of TLS inducers, leveraging both dendritic and stromal cells to reconstruct immune niches. Engineered DCs expressing CCL21, IL‐36γ, T‐bet, or IL‐15 boost antigen presentation and local immune activation, facilitating TLS formation in models of sarcoma, melanoma, and colon cancer [222, 223, 224, 225]. Clinical trials using adenoviral (Ad)‐CCL21‐DC in non‐small cell lung carcinoma patients demonstrated increased CD8^+^ T cell infiltration, suggesting potential synergy with immune checkpoint blockade [223]. Stromal cell transplantation using LN‐derived fibroblastic reticular cells or thymic stromal cells engineered to express LTα has led to TLS‐like structures with FDC networks, HEVs, and B/T cell compartmentalization in subcapsular tissues [226, 227]. The ability to therapeutically manipulate TLS formation to reduce disease severity, leading to improved prognoses and disease outcomes, proves to be a valuable medical tool. However, due to the complex heterogenous relationship between TLSs and different disease states, it is important to remain cognizant that enhancing TLS formation in some contexts may pose the risk of exacerbating other diseases, placing a heavy emphasis on how the risks and benefits are considered in each context.

### 3.2 Dismantling Pathological TLS

In contrast to their protective roles in infection or cancer, TLSs may exacerbate pathology in autoimmune and chronic inflammatory conditions, making their suppression a therapeutic goal in select contexts. However, in human patients, it remains unclear whether immunotherapeutic treatment disrupts TLS formation and structure.

Antibody‐based strategies have been explored to target key immune cell subsets involved in TLS generation. CD4^+^ T_H_17 cells, producers of IL‐17 and IL‐22, are frequently enriched in TLSs and drive inflammation in tissues such as the myocardium and kidneys. Antibodies targeting IL‐17 or podoplanin reduced TLS formation and pro‐inflammatory cytokine production in viral myocarditis and ischemia–reperfusion injury [228, 229, 230]. In autoimmune models like Sjögren's syndrome, IL‐17 blockade limited T and B cell infiltration, while IL‐27 was shown to suppress GC formation, potentially via FDC expansion [201]. Tfh cells, key producers of CXCL13 and IL‐21, can also be targeted to attenuate TLS formation and fibrosis in chronic kidney disease [204]. Antagonizing CXCL13 or blocking its receptor, CXCR5, disrupted TLSs in rheumatoid arthritis and diabetes models, highlighting its central role in maintaining ectopic lymphoid structures [231, 232, 233].

Pharmacological inhibitors provide another route to suppress TLS activity. Although direct in vivo evidence is lacking, recent mechanistic studies suggest that the Syk–Mcl‐1 signaling axis may be a promising therapeutic target for modulating B cell activity in TLS‐associated pathologies. In patients with chronic antibody‐mediated rejection, ectopic lymphoid structures resembling GCs form within the kidney, with infiltrating B cells expressing elevated levels of the anti‐apoptotic protein Mcl‐1. In vitro, the Syk inhibitor BAY61‐3606 reduced Mcl‐1 levels and impaired survival, activation, and IgG secretion of human tonsillar B cells [205]. FTY720 (fingolimod), an S1P receptor modulator, decreased B220^+^ B cells and CD4^+^ T cells in kidneys, leading to reduced TLS volume in ischemic models [229]. Interestingly, in non‐obese diabetic mice, FTY720 was found to preserve TLS structures in the pancreas, further highlighting the context‐dependence and heterogeneity of TLS responses [193].

In parallel, decoy receptor fusion proteins, such as LTβR‐Ig, can disrupt LTα1β2–LTβR signaling, a core axis in TLS assembly and organization. In experimental autoimmune encephalomyelitis models, systemic or local administration of LTβR‐Ig reduced CNS infiltration by B and T cells, suppressed expression of inflammatory chemokines CXCL13 and CXCL10, and prevented the development of meningeal B cell follicles [234]. Similarly, in primary Sjögren's syndrome, Baminercept (LTβR‐Ig) altered peripheral B and T cell dynamics and decreased plasma CXCL13 levels, although without clear clinical benefit or evidence that TLS are dismantled [235].

Targeting immunometabolism has emerged as a novel strategy to destabilize TLSs. In the K/BxN arthritis mouse model, inhibition of glycolysis with 2‐deoxyglucose significantly impaired Tfh cell expansion and reduced TLS‐associated inflammation and autoantibody production [236]. Similarly, blocking triglyceride synthesis in experimental autoimmune uveitis with A922500 dampened T_H_17 responses and ameliorated disease [237, 238]. In a broader context, enzymes like ACAT1 [239] and tryptophan metabolizing pathways [17] (known to regulate TLS formation and maturation in cancer) could represent therapeutic targets in autoimmunity as well. These findings suggest that metabolic interventions may offer a promising strategy to dismantle TLS and limit pathological immune activation in autoimmune diseases.

## 4 Future Directions in TLS Research

**FIGURE 6:**
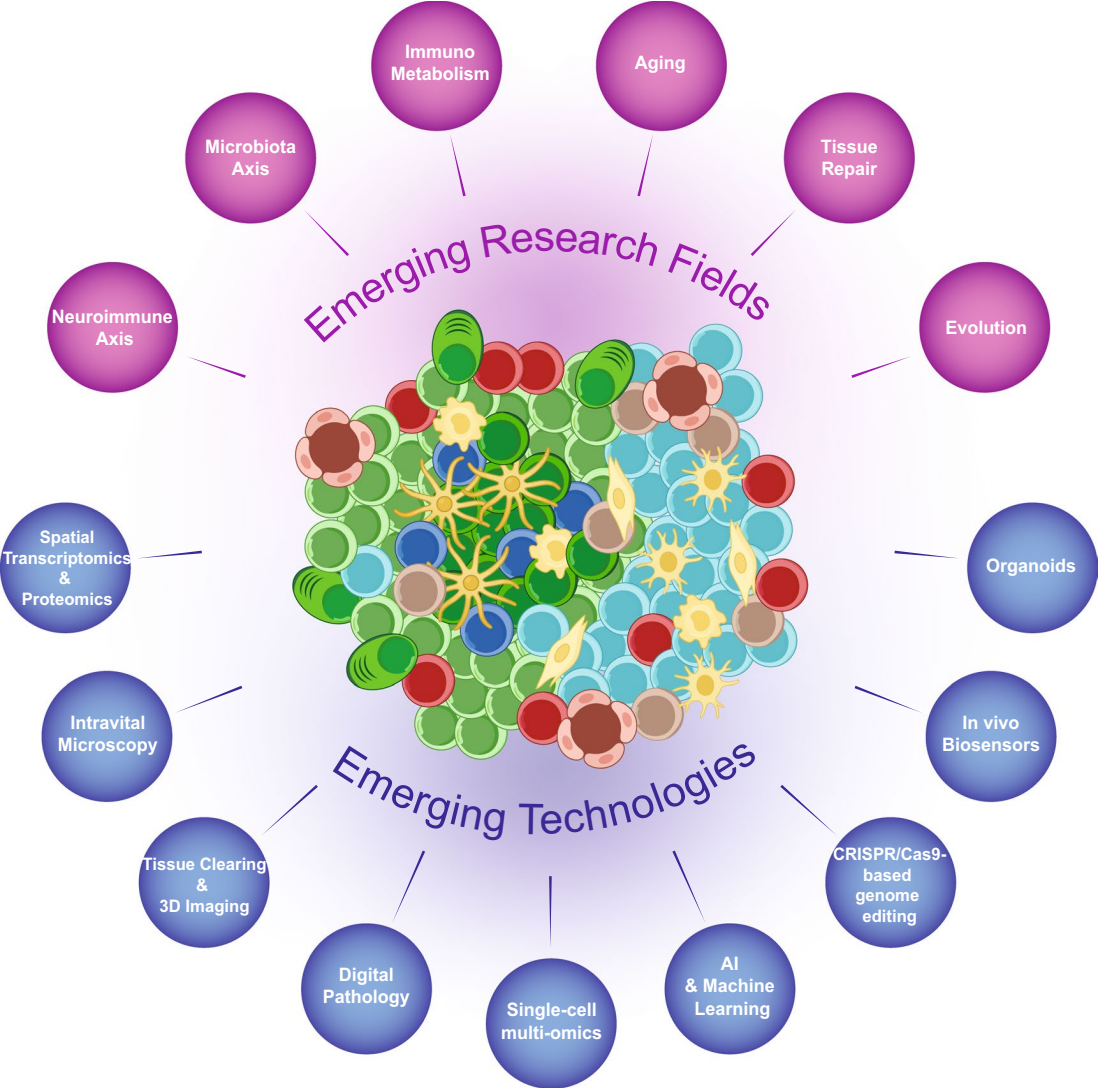
Emerging research directions and technologies in TLS biology. Conceptual overview of interdisciplinary fields and technologies advancing TLS research. Highlighted are emerging themes such as the neuroimmune and microbiota‐TLS axes, immunometabolism, tissue repair, aging, and evolutionary perspectives, alongside cutting‐edge tools including spatial transcriptomics, organoids, biosensors, AI, digital pathology, intravital imaging, and genome editing platforms.

TLS research is entering an exciting phase. Far from being uniform, TLSs exhibit contextual variation, which shapes the TLS to the tissue in which it forms, subject to the local structural, metabolic, and cellular constraints. To function effectively, TLSs form within unique environments in response to local tissue‐specific cues that orchestrate specialized immune responses. Simultaneously, the rapid development of innovative technologies is expanding our capacity to decode TLS biology with higher resolution. Together, these conceptual and technological advances are poised to redefine our understanding of TLSs, which may lead to new therapeutic avenues (Figure 6).

### 4.1 Active and Emerging TLS Research Fields

#### 4.1.1 Neuroimmune‐TLS Axis

Research suggests that the nervous system may play an important role in modulating TLSs through neuroimmune interactions. Notably, vagus nerve activity regulates TLS development in the inflamed colon, with its ablation reducing CXCL13 expression and impairing TLS formation [161]. Similarly, sympathetic nervous system involvement is evident, as the absence of sympathetic innervation hampers TLS generation upon acute lung inflammation [240]. These findings underscore the significance of neural inputs in modulating TLSs, suggesting potential avenues for therapeutic interventions targeting neuroimmune pathways in diseases characterized by chronic inflammation and TLS formation.

#### 4.1.2 Microbiota‐TLS Axis

The human microbiome has a key role in immune development, tissue homeostasis, and disease progression [241, 242, 243]. Among its many functions, the microbiota has also emerged as a potential modulator of TLSs, particularly in mucosal and immune‐active tissues like the gut, lungs, and liver, organs closely linked through the bidirectional gut–lung axis. Recent studies have also revealed a compelling link between skin microbiota and TLS induction in the skin [244]. Microbiome‐derived signals, including short‐chain fatty acids, bacterial antigens, metabolites, and bile acids, further influence both stromal and immune cell compartments [43, 171]. In contrast, dysbiosis can impair TLS function, contributing to conditions such as inflammatory bowel disease, colorectal cancer, and tumor‐associated immunosuppression [245, 246, 247, 248]. The extensive microbiome diversity between individuals likely contributes to the heterogeneity of gut TLSs. These insights underscore the need to explore the microbiota–TLS axis within organ‐specific contexts, particularly where microbial‐immune interactions are most dynamic.

#### 4.1.3 TLS and Immunometabolism

TLSs arise in metabolically demanding environments, often persisting under hypoxic or nutrient‐deprived conditions. Hypoxia‐inducible factors (e.g., HIF‐1α) and metabolic pathways such as glycolysis and fatty acid oxidation regulate both stromal and lymphocyte function within TLSs [244]. Since mature TLSs frequently host functional GC‐like reactions, understanding the metabolic constraints of these niches across tissues is crucial for targeted intervention. GCs themselves are highly dynamic and hypoxic, shaping both B cell and Tfh cell behavior [249]. GC B cells are metabolically unique among proliferating lymphocytes, relying on fatty acid oxidation for ATP production while exhibiting minimal glycolysis for energy [250, 251]. Evidence suggests that zones of the GC, particularly the light zone, may be hypoxic. Indicator dyes and the expression of hypoxia‐inducible factors HIF‐1 and HIF‐2 in GC B cells support this idea [252], however, the specificity of these probes is uncertain, emphasizing the need for further anatomical and functional studies of GC vasculature and oxygen dynamics. In tumors, metabolic reprogramming, including nutrient scarcity, altered amino acid metabolism, and tryptophan accumulation, can impair TLS maturation and GC formation, dampening antitumoral immunity [17, 253]. These findings underscore the therapeutic promise of targeting immunometabolism to modulate TLSs, either by promoting their formation to convert cold tumors into immunotherapy‐responsive environments [253], or by suppressing them in autoimmune diseases where TLSs sustain chronic inflammation and tissue damage.

#### 4.1.4 TLS and Aging

Age‐associated TLS arise spontaneously in several tissues, including the liver and kidney, independent of infection or overt inflammation [32, 121, 254] and are often associated with senescent B and T cells, altered cytokine profiles, and chronic low‐grade inflammation. Whether such TLS are protective, neutral, or deleterious remains an open question but will become increasingly pertinent with aging populations worldwide.

#### 4.1.5 TLS in Tissue Repair and Regeneration

In wound healing and fibrotic diseases, TLS formation can either support tissue remodeling or perpetuate chronic inflammation. TLS have been identified in skin, lung, and liver during repair phases, where they may orchestrate local immune responses to injury or facilitate resolution [1, 4, 33, 203]. Fibroblast heterogeneity also shapes TLS dynamics, with some subsets promoting fibrosis and others supporting immune cell recruitment and TLS organization, an effect particularly evident in age‐dependent renal fibrosis and inflammation [191, 255]. Moreover, TLSs are frequently found in fibrotic regions in autoimmune diseases, where they have been functionally linked to the promotion and maintenance of fibrosis, further complicating their role in tissue repair [200]. More evidence is required to understand the complexity of tissue damage and repair associated with TLS.

#### 4.1.6 Comparative Evolution

Comparative evolutionary studies of TLSs can offer valuable insights into their fundamental role across species. While well‐described in mammals, similar lymphoid aggregates have been discovered in species that lack LNs, such as in birds [256, 257, 258], reptiles, and fish, suggesting that ectopic immunity is an adaptive response to chronic inflammation or persistent antigens [259, 260, 261, 262]. These structures likely evolved prior to the existence of conventional lymphoid organs to provide localized immune function by supporting local affinity maturation and directly seeding local tissue‐resident memory B cell populations [79]. Studying TLS‐like formations across organisms and organs may shed light on their core functions and evolutionary raison d'être as hubs for immune coordination, protection, and repair in response to environmental challenges.

### 4.2 Emerging Technologies to Investigate TLS

Technological innovations are revolutionizing how TLSs are studied, enabling a shift from descriptive histology to dynamic, high‐resolution analysis. Spatial transcriptomics and proteomics now allow in situ mapping of RNA and protein expression across TLS microdomains, uncovering spatially organized gene networks and cell–cell interactions [17, 19, 263, 264]. Intravital microscopy provides real‐time visualization of immune cell trafficking, GC formation, and structural changes during TLS development, particularly in murine models of infection, inflammation, and cancer [265, 266]. Complementary to this, 3D imaging via tissue clearing and light‐sheet microscopy enables comprehensive visualization of TLS architecture at the whole‐organ level, especially within lung, liver, and brain tissues [267, 268]. Digital pathology powered by machine learning and medical imaging techniques, including computed tomography, positron emission tomography, single‐photon emission computed tomography, and magnetic resonance imaging, is becoming integral for the standardized identification and maturity scoring of TLSs in clinical biopsies, facilitating their use as prognostic and predictive biomarkers [269]. Single‐cell multi‐omics, combining transcriptomics, chromatin accessibility, and proteomics, now enables fine‐scale dissection of TLS cellular heterogeneity, lineage trajectories, and clonal relationships [270]. Recent advances in spatial B cell receptor sequencing are beginning to enable the mapping of somatic hypermutation and affinity maturation within TLS‐associated GCs in human tissues, offering new insights into local antibody evolution and selection pressures [271, 272]. CRISPR‐based screening, applied in vivo or in TLS‐resembling organoids, is a powerful approach to uncover genetic regulators of TLS formation, such as key transcription factors, cytokine receptors, or metabolic enzymes [273, 274]. Genetically encoded and synthetic biosensors are being designed to monitor TLS‐local conditions like oxygen tension, pH, or cytokine gradients, allowing direct correlation between microenvironmental cues and TLS behavior [275]. Finally, new organoid models and ex vivo systems recapitulating TLS‐like microenvironments could offer human‐relevant platforms to dissect TLS ontogeny, test therapeutics, and explore tissue‐specific immunological circuits [274, 276]. Together, these technologies are enabling researchers to unravel the dynamic and context‐dependent roles of TLSs with unprecedented clarity, fostering novel diagnostic and therapeutic strategies across diseases.

## 5 Concluding Remarks

TLSs have emerged as dynamic, context‐sensitive immune niches capable of supporting GC‐like activity outside canonical SLOs. As detailed in this review, TLSs can spontaneously form in a wide variety of tissues, including the lung, liver, skin, CNS, and vasculature, each with unique microenvironmental cellular structures and metabolic cues that impact and regulate the formation of TLSs. These variations are shaped by local tissue‐specific signals, ranging from stromal microarchitecture and metabolic constraints to microbial exposure and neural innervation, underscoring the importance of tissue context in TLS biology.

TLSs play divergent roles depending on the inflammatory and stimulating contexts. In many infections and cancers, they can support protective immune responses and local memory formation. Conversely, in autoimmune and chronic inflammatory disease states, they may promote pathology through sustained immune activation and autoantibody production. While the dual nature of TLS modulation presents both therapeutic promise and complexity, emerging biomedical and technological advances are opening new avenues to exploit TLSs in immunotherapy.

Recent advances in spatial transcriptomics, imaging, biomaterials, and cellular engineering have expanded our ability to understand the nature of TLSs, but many key conceptual challenges persist:

– What is the contribution of TLSs to local versus systemic immune responses, and how do they inform our understanding of immune system decentralization?
– Do TLSs serve as incubators for organ‐specialized immune cells, and can these cells provide superior protection tailored to their tissue of origin?
– Is there a unique form of immunological imprinting or clonal evolution that occurs within TLSs, distinct from that in canonical lymphoid organs, and does this imprinting shape organ‐tropic memory T and B cell populations?
– To what extent do stromal–immune cell interactions influence the selection, differentiation, and fate of emerging immune clones within TLSs?
– If architectural organization is critical for TLS function, what defines the minimal structural requirements, and to what extent are these requirements shaped by tissue‐specific contexts?
– How can we better tailor medical interventions and drug design to selectively manipulate tissue‐specific TLSs for improved disease progression and prevention?

Addressing these questions will not only deepen our mechanistic understanding of TLS biology but also reveal new therapeutic opportunities across a range of diseases. Rather than viewing TLSs as aberrant or redundant lymphoid outposts, it is time to recognize them as integral, highly adaptive components of immune architecture, poised at the intersection of tissue identity, immune function, and clinical relevance.

## Conflicts of Interest

M.I. consults for, receives grants from, or sits on advisory boards of Gilead Sciences, GentiBio, BioNTech, GSK, Curie Bio. M.A.L. has recieved funding from GSK outside of this work.

## Data Availability

Data available on request from the authors.
